# Danlou Tablets Inhibit Atherosclerosis in Apolipoprotein E-Deficient Mice by Inducing Macrophage Autophagy: The Role of the PI3K-Akt-mTOR Pathway

**DOI:** 10.3389/fphar.2021.724670

**Published:** 2021-09-08

**Authors:** Chunping Liu, Guiling Chen, Yanfen Chen, Yue Dang, Guangning Nie, Dinghong Wu, Jinhua Li, Zide Chen, Hailong Yang, Dongyue He, Xiong Li, Jingbo Sun, Jiahong Lu, Lei Wang

**Affiliations:** ^1^ State Key Laboratory of Dampness Syndrome of Chinese Medicine, The Second Affiliated Hospital of Guangzhou University of Chinese Medicine, Guangzhou, China; ^2^ Department of Cardiovascular Medicine, Guangdong Provincial Hospital of Chinese Medicine, Guangzhou, China; ^3^ State Key Laboratory of Quality Research in Chinese Medicine, Institute of Chinese Medical Sciences, University of Macau, Macau, China; ^4^ Department of National Institute of Stem Cell Clinical Research, Guangdong Provincial Hospital of Chinese Medicine, Guangzhou, China; ^5^ Puning Hospital of Traditional Chinese Medicine, Puning, China; ^6^ College of Traditional Chinese Medicine, Shenyang Pharmaceutical University, Shenyang, China; ^7^ Department of Interventional Radiology, Cancer Center, Guangdong Provincial People’s Hospital, Guangdong Academy of Medical Sciences, South China University of Technology, Guangzhou, China; ^8^ Guangdong Provincial Key Laboratory of Research on Emergency in TCM, Guangzhou, China

**Keywords:** atherosclerosis, danlou tablets, apolipoprotein E deficient mice, macrophage, autophagy, PI3K/Akt/mTOR signaling pathway

## Abstract

Atherosclerosis (AS) is a type of chronic vascular disease, and its etiology is not yet fully understood. AS is characterized by lipid deposition, atherosclerotic plaque formation, vascular stenosis or even complete blockage of the blood vessel wall. Clinical studies have shown that Danlou tablets (DLTs) can improve the heart function, quality of life, and prognosis of patients with coronary heart disease and myocardial infarction. However, its mechanism of action remains unknown. Our study revealed that DLTs ameliorated ApoE^−/−^AS mouse aortic atherosclerotic plaques [hematoxylin-eosin (HE) staining and small animal ultrasound] and reduced CD68^+^ macrophage infiltration, the expression of the inflammatory factor interferon-gamma (IFN-γ), vascular smooth muscle α-actin, and serum lipid levels. *In vitro*, in the macrophage foaming model, DLTs partially restored the activity of RAW264.7 cells, reduced the uptake of lipid droplets, and inhibited lipid droplet accumulation and apoptosis within BMDMs. We also found that Torin1, an autophagy agonist, reduced intracellular lipid deposition in BMDMs, as did DLTs. Moreover, DLTs upregulated the expression of the autophagy-related protein LC3II and decreased p62 accumulation in RAW264.7 cells. DLTs also inhibited the phosphorylation of p-PI3K, p-Akt, and p-mTOR, leading to upregulated autophagy in RAW264.7 cells. In summary, our results suggested that DLTs can promote autophagy in macrophages by inhibiting the PI3K/Akt/mTOR signaling pathway, thereby reducing foam cell formation and improving atherosclerosis.

## Introduction

Atherosclerosis is a progressive disease characterized by the accumulation of lipid-rich macrophages on the inner wall of the artery, accompanied by the proliferation of vascular smooth muscle cells and fibrous matrix, forming asymmetric focal thickening of the intima, and gradually developing into the formation of atherosclerotic plaques. Arterial plaque can cause vascular stenosis or even complete blockage and ultimately cause ischemia or infarction of the corresponding organ. In addition, atherosclerotic plaques rupture and trigger thrombosis (Stary, Chandler et al., 1995; Nanchen and Raggi 2017). At present, the treatments of atherosclerosis mainly depend on statins, antithrombotic drugs and surgical intervention. Statins are currently the most effective drugs to prevent and treat cardiovascular events (Horodinschi, Stanescu et al., 2019). However, high-dose statin administration has been associated with severe side effects, including an increased risk of new-onset diabetes, liver damage, rhabdomyolysis, etc.

In China, a proprietary medicine named Danlou tablets (DLTs) has long been considered a common therapy for cardiovascular diseases (Mao, Wang et al., 2016). DLTs have the characteristics of good curative effects, inducing an obvious improvement of symptoms, and mild adverse reactions, which have been widely recognized in long-term clinical practice. DLTs are composed of 10 types of Chinese herbs: *Trichosanthes kirilowii Maxim. [Cucurbitaceae; Pericarpium Trichosanthis]; Allium macrostemon Bge [Liliaceae; Bulbus allii macrostemonis] Pueraria lobata (Willd.) Ohwi [Leguminosae; Radix puerariae]; Ligusticum chuanxiong hort [Umbelliferae; Rhizoma chuanxiong]; Salvia miltiorrhiza Bunge [Lamiaceae; Salviae miltiorrhizae radix et rhizoma]; Paeonia lactiflora Pall [Ranunculaceae; Radix paeoniae rubra]; Alisma orientalis (Sam.) Juzep [Alismataceae; Rhizoma alismatis]; Astragalus membranaceus (Fisch.) Bge [Leguminosae; Radix astragali]; Drynaria fortunei (Kunze) J. Sm.[Davalliaceae; Rhizoma drynariae] and Curcuma wenyujin Y.H. Chen et C. Ling [Zingerberaceae; Radix curcumae]*. Randomized controlled trials of 219 patients undergoing percutaneous coronary intervention (PCI) with non-ST elevation acute coronary syndrome (NSTE-ACS) showed that the DLT group could reduce the incidence of periprocedural myocardial infarction in patients with ACS undergoing PCI. The incidence of major adverse cardiac events (MACEs) at the 90 day follow-up was significantly decreased in the DLT group compared with the placebo group, and the incidence of nonfatal myocardial infarction at 90 days was reduced in the DLT group (Wang, Zhao et al., 2016). *In vivo*, several studies have shown that DLTs can improve dyslipidemia and atherosclerosis (Liu, Lin et al., 2013; Gao, Xue et al., 2020; Tang, Li et al., 2020). In apolipoprotein E (ApoE)-knockout mouse models of myocardial ischemia, DLTs could regulate the NF-κB signaling pathway related to inflammatory factors, including TNF-α, IL-6, IL-1β, IL-8, MMP-1 and MMP-2, to alleviate atherosclerosis (Gao, Xue et al., 2020). Tang et al. predicted that DLTs can improve the TG and TC levels of ApoE^−/-^ mice treated with chronic intermittent hypoxia through the HIF-1α-Angptl4 mRNA signaling pathway, as well as the area of atherosclerotic plaques (Tang, Li et al., 2020).

Macrophages play important roles in all stages of atherosclerosis, including plaque formation and rupture. In the early stage, oxidized LDL (ox-LDL), aggregated lipoproteins, and other substances are internalized by macrophages, which transform into foam cells. These foam cells enter the inner membrane and release inflammatory mediators to increase the permeability of the endothelium and damage the cells and mononuclear macrophages, thus forming a vicious cycle and inducing the progression of atherosclerosis. A study by Deng et al. indicated that ethanol extracts of DLTs attenuate atherosclerosis through anti-inflammation and prevent lipid deposition in macrophages by suppressing NF-κB signaling and triggering the PPARα/ABCA1 signaling pathway (Hao, Danbin et al., 2019).

Autophagy is a self-protective process mediated by the degradation and recycling of cytoplasmic products to maintain cell homeostasis. Autophagy has been proven to be involved in lipid metabolism, termed macrolipophagy, and is considered a protective mechanism during atherosclerosis (Martinet and De Meyer 2009; Singh, Kaushik et al., 2009; Sergin, Evans et al., 2017; Miao, Zang et al., 2020). Impaired autophagy accelerates atherosclerosis by regulating the dysfunction of lipid metabolism in macrophages (Li W. et al., 2016; Evans, Jeong et al., 2018).

Although DLTs are commonly used for patients with coronary heart disease in China, their mechanism of action has not yet been elucidated. In this study, we used an ApoE^−/-^ mouse model and *in vitro* cell lines, including RAW264.7 cells and BMDMs, to explore the mechanisms of DLTs in treating atherosclerosis. The findings indicated that DLTs improved atherosclerosis by enhancing autophagy in macrophages.

## Materials and Methods

### Ultrahigh-Performance Liquid Chromatography Analysis

Five different batches of DLTs were monitored for quality control by UHPLC. Briefly, DLTs and 4 standards (danshensu; salvianolic acid A; cryptotanshinone; tanshinone ⅡA) were dissolved in methanol-0.1% formic acid. Chromatographic separation was performed using an Accela™ UHPLC system, which was comprised of a UHPLC pump and a PDA detector with scanning from 200 to 400 nm and recorded at 214 nm. The HPLC conditions were set as follows: column: KintexR C18, 150 mm × 2.1 mm, 2.6 μm particle size (Phenomenax, United States); mobile phase components: A was water with 0.1% formic acid and B was methanol; flow rate: 250 μl/min; injection volume: 10 μl; gradient: 0–45 min, linear gradient of 10–35% A, 45–50 min, 35–46% A, 50–60 min, 46–85% A.

### Ethics Statement

All experimental animals were treated responsibly and humanely, and all animal experiments and breeding programs complied with the Animal Handling Regulations of the People’s Republic of China (Ministry of Health, People’s Republic of China, Regulation No.: 552001). The protocol was approved by the Ethics Committee at Guangdong Provincial Hospital of Chinese Medicine and was conducted in accordance with the 1989 Declaration of Helsinki.

### Animals and Models

ApoE^−/-^ mice (male, 8 weeks) were purchased from Vital River Laboratory Animal Technology Co., Ltd. (Beijing, China) and housed in specific pathogen-free (SPF) environments in climate-controlled conditions (20–23°C, 45–65% humidity) and illumination (12 h light/dark cycles) with access to food and water ad libitum. The light period was 7 am to 7 pm, and adaptive feeding was performed for 7 days before the experiment. ApoE^−/-^ mice were randomly divided into six groups: the control group; the model group; the DLT-low group (120 μg/kg); the DLT-middle group (240 μg/kg); the DLT-high group (480 μg/kg) (provided by Connell Pharmaceutical Co., Ltd. [Jilin, China], batch number: 20191204) (Lin, Wang et al., 2020); and the atorvastatin group (2 mg/kg) (purchased from Pfizer [United States]). The control and model groups were administered the same volume of 0.9% normal saline (normal saline 1.5 ml/kg/d) every day. All treatments were administered orally by gavage. The control group was fed a normal diet for 16 weeks; the model group and other groups were fed a high-fat diet for 16 weeks. There were *n* = 7 mice in each group.

### Hematoxylin-Eosin Staining and Oil Red O Staining of Tissue Slices

Hematoxylin-eosin (HE) staining and oil red O staining were conducted to evaluate left carotid or aortic structure atherosclerosis. Tissue isolated from all groups was fixed (4% paraformaldehyde for 24 h), dehydrated, transparentized, waxed, embedded, sectioned, and then stained with HE and oil red O.

### High-Frequency Ultrasound

Sonographic evaluation of carotid arteries was conducted using a high-resolution ultrasound device (Vevo2100, FUJIFILM VisualSonics Inc.) and a high-frequency transducer probe (VisualSonics MS-550D, FUJIFILM VisualSonics Inc.). The transducer probe with a frequency of 22–50 MHz provided a characteristic resolution of 13.9 µm and a maximum imaging depth of 0–10 mm.

### Immunohistochemistry

The paraffin tissue sections were dewaxed and then treated with 0.3% hydrogen peroxide solution for 10 min. Then, the sections were immersed in 0.01 mol/L citric acid buffer and heated to boiling in an autoclave 3 min 3 times. Next, the sections were blocked with 5% bovine serum albumin (BSA) for 1 h at room temperature. Then, the samples were washed three times with phosphate-buffered saline (PBS) and incubated with primary antibodies (rat anti-mouse α-actin [dilution 1:100], rabbit anti-mouse IFN-γ [dilution 1:100] and rabbit anti-mouse CD68 [dilution 1:50], all purchased from Abcam, United Kingdom) for 1 h at 37°C. The sections were then rinsed three times with PBS, treated with appropriate horseradish peroxidase (HRP)-conjugated secondary antibodies for 30 min at 37°C and washed 3 times with PBS again. Antigen-antibody reactions were stained with diaminobenzidine (DAB) for 5–10 min, and all sections were counterstained with hematoxylin. The expression levels of CD68, IFN-γ and α-actin were observed with a Nikon E400 microscope under high-power (400×) fields.

### Real-Time PCR

Total RNA was extracted from fresh frozen aortae with a FastPure Cell/Tissue total RNA isolation kit (Vazyme, China) according to the manufacturer’s instructions. Total RNA (1 μg) was reverse transcribed to cDNA with a HiScript III RT SuperMix for use in a qPCR kit (Vazyme, China) at 37°C for 15 min and 85°C for 5 s. Extracted mRNA was then reverse transcribed into cDNA using the iScript cDNA synthesis kit (Bio-Rad, United States). Real-time PCR analysis was performed using ChamQ Universal SYBR qPCR master mix (Vazyme, China) and an Applied Biosystems Quant Studio 3 Detection System according to the manufacturer’s recommendations (Thermo Fisher Scientific, United States). Specific primers for IFN-γ, TNF-α, oxidized low-density lipoprotein receptor 1 (LOX-1), IL-10, PI3K, AKT and mTOR were designed using Primer 3 software (Table 1). A melting curve analysis was performed to confirm the specificity of qPCR products. Fold-changes were calculated using the 2^−∆∆Ct^ method and were normalized to β-actin expression. All reactions were performed in triplicate and repeated three times.

**TABLE 1:** qPCR primer sequences used in this study.

Target gene	Primer sequence (forward)	Primer sequence (reverse)
IFN-γ	AGTGGCATAGATGTGGAA	CAATGACGCTTATGTTGT
TNF-α	CCC​CAG​TCT​GTA​TCC​TTC​TAA	TCGAGGCTCCAGTGAATT
LOX-1	TCT​TCC​ATG​GGC​CCT​TTA​GCT​G	TTC​CGA​TGC​AAT​CCA​ATC​CAG​A
IL-10	ACC​TGC​TCC​ACT​GCC​TTG​CT	GGT​TGC​CAA​GCC​TTA​TCG​GA
PI3K	CTT​GCC​TCC​ATT​CAC​CAC​CTC​T	GCC​TCT​AAT​CTT​CTC​CCT​CTC​CTT​C
AKT	TGT​CTC​GTG​AGC​GCG​TGT​TTT​T	CCG​TTA​TCT​TGA​TGT​GCC​CGT​C
mTOR	AGT​GAA​GCC​GAG​AGC​AAT​GAG​A	GCC​AAG​GAG​ATA​GAA​CGG​AAG​AAG​C
β-actin	AAC​ACC​ACC​CAG​TTG​CTG​TA	TCC​ACC​ACC​CAG​TTG​CTG​TA

### Serum Chemistry Analysis

All ApoE^−/-^ mice were fasted overnight before being anesthetized and sacrificed. All blood samples were placed at 4°C overnight and then centrifuged at 3,000 rpm for 10 min to obtain the upper serum. Total cholesterol (TC), triglycerides (TGs), high-density lipoprotein cholesterol (HDL-C) and low density lipoprotein cholesterol (LDL-C) were measured by an automatic animal biochemistry analyzer (Catalyst One, IDEXX, United States).

### Network Target Prediction

First, the compounds meeting the Lipinski “rule of five” and drug-likeness (DL) ≥0.18 criteria in DLTs were searched using 3 databases: the Lab of Systems Pharmacology database; the TCM Database @ Taiwan; and BATMAN-TCM. Autophagy-related targets were obtained by using known autophagy-related targets from two existing databases: the TTD database (http://database.idrb.cqu.edu.cn/TTD/) and the DrugBank database (http://www.drugbank.ca/). Then, we used MOE software for molecular docking of the compounds to the major autophagy-related targets. The component-target network was established using Cytoscape 3.6.1 software (Bethesda, MD, United States).

### Cell Culture and Treatment

RAW264.7 macrophages (Xiangya Central Laboratory Cell Bank, Central South University, Changsha, China) were cultured in high-glucose Dulbecco’s modified Eagle’s medium (DMEM) (HyClone, United States) supplemented with 10% heat-inactivated fetal bovine serum (FBS) (Gibco, United States), 100 U/mL penicillin, and 100 μg/ml streptomycin (Gibco, United States) at 37°C in a humidified incubator containing 5% CO_2_.

Mouse BMDMs were obtained by flushing the femurs of C57BL/6 mice and cultured with DMEM containing 10% heat-inactivated FBS. To obtain differentiated macrophages, 5 ng/ml macrophage colony-stimulating factor (R&D Systems) was added every 2 days for 7 days.

After 24 h of seeding in a 6-well plate (3506, Corning, United States), these cells were randomly divided into different groups: control; Torin1; Model (oxLDL); DLT^low^ (7.5 μg/ml); DLT^middle^ (75 μg/ml) and DLT^high^ (750 μg/ml).

### MTT Assay

The effect of DLTs (Connell, Jilin, China) on RAW264.7 macrophage viability was assessed by measuring the absorbance of 3-(4.5-dimethylthiazol-2-yl)-2, 5-diphenyl tetrazolium bromide (MTT) dye in the cells. RAW264.7 cells (5×10^3^, 4×10^3^ and 3×10^3^) were seeded in 96-well culture plates (Corning, United States) and treated with DLTs (0, 30, 60, 90, 120, 180, 210, 240, 270 and 300 μg/ml) for 24, 48, and 72 h, respectively. Subsequently, 100 µl of MTT solution (0.5 mg/ml) was added to each well and incubated at 37°C for another 4 h. Then, the supernatant was discarded and replaced with 100 μl of DMSO. The optical density was read on a PerkinElmer VICTOR X5 (PerkinElmer, United States) with a monochromatic microplate reader at a wavelength of 490 nm.

### Cell Index Evaluation Using the xCELLigence System

Experiments were performed using the xCELLigence real-time cell analysis (RTCA) DP instrument (ACEA Biosciences Inc., San Diego, CA, United States) at 37°C with 5% CO_2_. To measure the CI of RAW264.7 cells on oxLDL and DLTs in real time, RAW264.7 cells were seeded on gold microelectrodes embedded at the bottom of 16-well microplates (E-plates; ACEA Biosciences Inc., San Diego, CA, United States) at a density of 7.0×10^3^ cells/well. After the baseline measurement was calibrated, the cell index was measured every 15 min. Then, 24 h after seeding, 50 μg/ml oxLDL and 12, 120, 210, 240, 270 and 300 μg/ml DLTs were added to the culture system every 1 min for 2 h and every 15 min for 249 h to observe the short-term and long-term effects of the drug, respectively.

### Phagocytosis of Dil-oxLDL by RAW264.7 Cells

RAW264.7 cells were inoculated in 24-well plates, 10 μg/ml DiI-oxLDL was added to induce the formation of foam cells, and different concentrations of DLTs were applied to intervene in the process. The control group was supplemented with culture medium without DiI-oxLDL or DLTs. Flow cytometry was used to analyze the content of DiI-oxLDL phagocytosed by the cells of each group by measuring the median fluorescence intensity (MFI).

### Oil Red O Staining of BMDMs

BMDMs were cultured in 6-well culture plates, and the corresponding drugs were added for 24 h of treatment. Cells were gently rinsed twice with PBS solution and fixed with 4% paraformaldehyde for 30 min. Cells were rinsed with PBS solution for 1 min, stained with oil red O working solution for 30 min at room temperature, and washed twice with distilled water for 1–2 min. Red intracellular lipid droplets were subsequently observed under a microscope.

### DAPI-PI Staining of BMDMs

BMDMs were seeded in 6-well plates. After treatment with DLTs and/or ox-LDL, cells were rinsed 2 times with PBS and then incubated with DAPI staining solution for 15 min at room temperature in the dark. The solution was discarded and stained with PI dye solution for 10 min after rinsing 2 times with PBS. Cells were observed under an immunofluorescence microscope.

### Western Blotting

RAW264.7 cell protein was extracted using RIPA buffer. Protein concentration was measured using the BCA Kit (Beyotime Biotechnology, China). The protein samples (10 µg/well) were separated by 10–15% sodium dodecyl sulfate (SDS)-polyacrylamide gel electrophoresis and then transferred to a polyvinylidene difluoride (PVDF) membrane (Millipore, United States). Each PVDF membrane was incubated with primary monoclonal antibodies against p-PI3K (17366), p-Akt (4060), p-mTOR (5536), Akt (4691), mTOR (2983), LC3 (12741), p62 (23214) and GAPDH (5174) (Cell Signaling Technology, United States) overnight at 4°C. Then, the cells were blotted with a secondary antibody conjugated with horseradish peroxidase for 1 h at a dilution of 1:5000. Finally, the bands were visualized by enhanced chemiluminescence staining. The intensities of the bands were quantified with ImageJ software. (NIH Image, United States)

### Statistical Analysis

Statistical analysis was performed using SPSS 17.0 (SPSS, Inc., United States). The data were expressed as the mean ± SD. When the data conformed to normality and homogeneity of variance, one-way ANOVA was used to analyze the data. Otherwise, the Kruskal-Wallis method was used for analysis. Values of *p* < 0.05 were considered significant.

## Results

### Chemical Profiles of Danlou Tablets

For the quality control of DLTs, we detected the main chemical compositions of DLTs by UHPLC analysis. UHPLC fingerprints of five batches of DLTs were obtained (Supplementary Figure S1 in the Supplementary Material).

### Danlou Tablets Reduced the Formation and Number of Atherosclerotic Plaques

**FIGURE 1:**
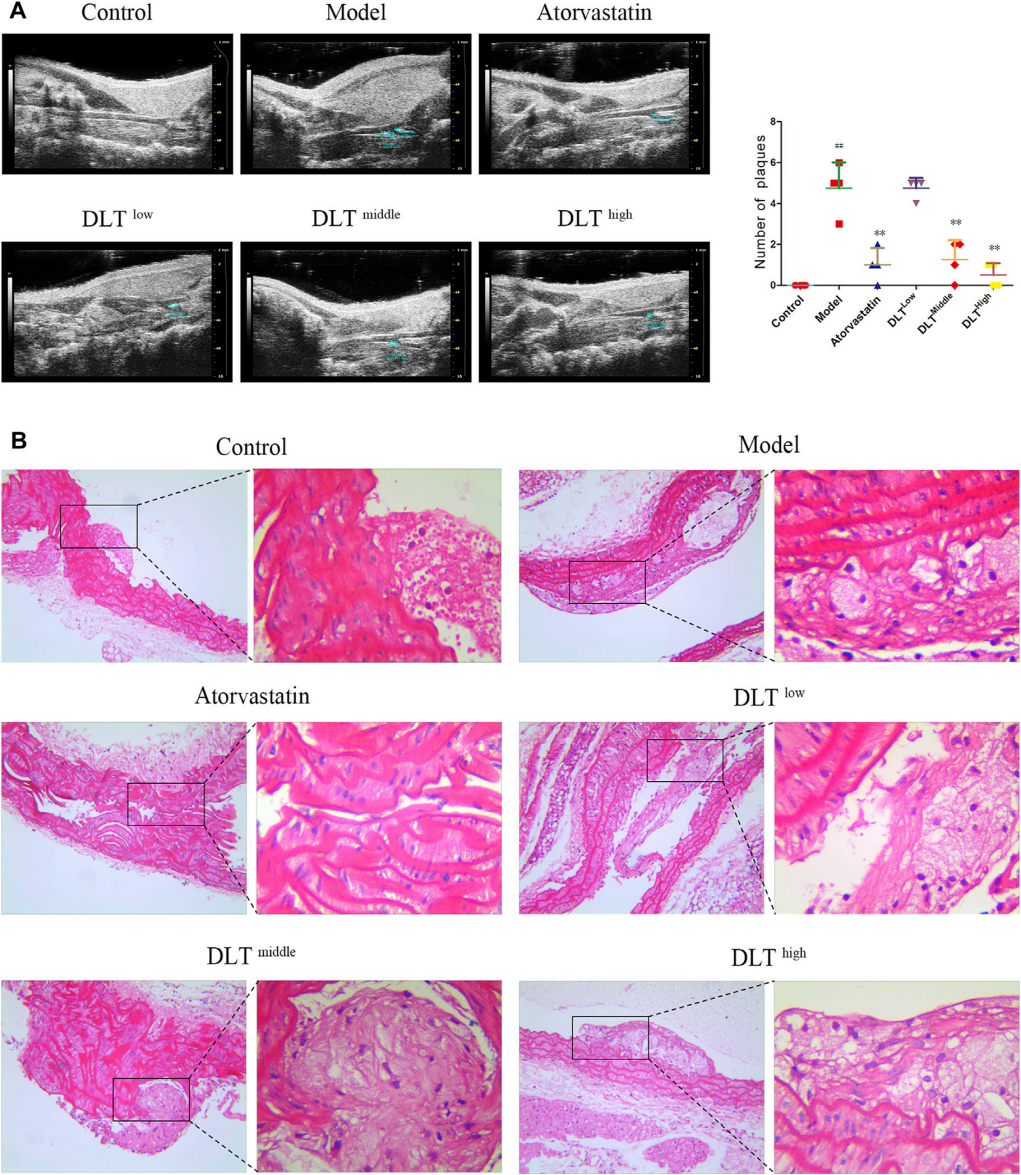
DLTs reduced atherosclerotic plaque formation of the aorta and carotid artery in ApoE^-/-^ AS mice. Plaques were observed by **(A)** vascular ultrasound of the carotid artery and **(B)** HE staining of aortic tissue sections. **(A)** The control group showed the uniformly continuous vascular acoustic shadow of the carotid artery, and no abnormal acoustic shadow was observed in the lumen; most mice in the model group had an uneven continuous sound shadow in the carotid artery, some of the vessel walls had a hyperechoic shadow, and an abnormal sound shadow was present in the vessel wall (plaques, see circled); inhomogeneous and continuous hyperechoic lights were also observed in the carotid arteries of the DLT-low group; the phenomenon was effectively restrained in DLT-middle and DLT-high groups and the atorvastatin group. The number of plaques is indicated in graphs. **(B)** In the model group, the endothelial layer was sloughed off, with more plaques attached to the vessel wall, intimal hyperplasia was severe, many foam cells could be observed; compared with the model group, the area of plaques in the DLT groups and the atorvastatin group was smaller, the foam cell infiltration was reduced and the effect was dose-dependent in the DLT groups. The high-dose DLT group and the atorvastatin group showed continuous vascular endothelial cells. The data are presented as the mean ± SD (*n* = 4). ^##^ *p* < 0.01 vs the control group, and ^**^ *p* < 0.01 vs the model group.

A small animal ultrasound machine was used to acquire and evaluate the effects of DLTs on atherosclerotic plaques in ApoE^−/-^ mice after 16 weeks with high fat diets (Figure 1A). The results revealed middle- and high-dose DLTs and atorvastatin reduced the number of atherosclerotic plaques compared with the model group (*p* < 0.01), whereas there is no significant difference between the low-dose DLT group and the model group in the number of plaques (*p* > 0.05). The ultrasound image in the control group showed smooth carotid arteries without plaque formation.

After HE staining, the plaque structure of atherosclerosis mice in each group was observed under a 200× optical microscope, as shown in Figure 1B. In the model group, the endothelial layer was sloughed off, with a large number of plaques attached to the inner wall of the vessel, severe intimal hyperplasia and massive foam cell infiltration. Compared with the model group, the area of plaques in the DLT groups and the atorvastatin group was smaller, the foam cell infiltration was reduced with a dose-dependent effect in the DLT groups. The high-dose DLT group and the atorvastatin group showed continuous vascular endothelial cells. HE staining results showed that DLTs can reduce plaque formation and foam cell infiltration.

**FIGURE 2:**
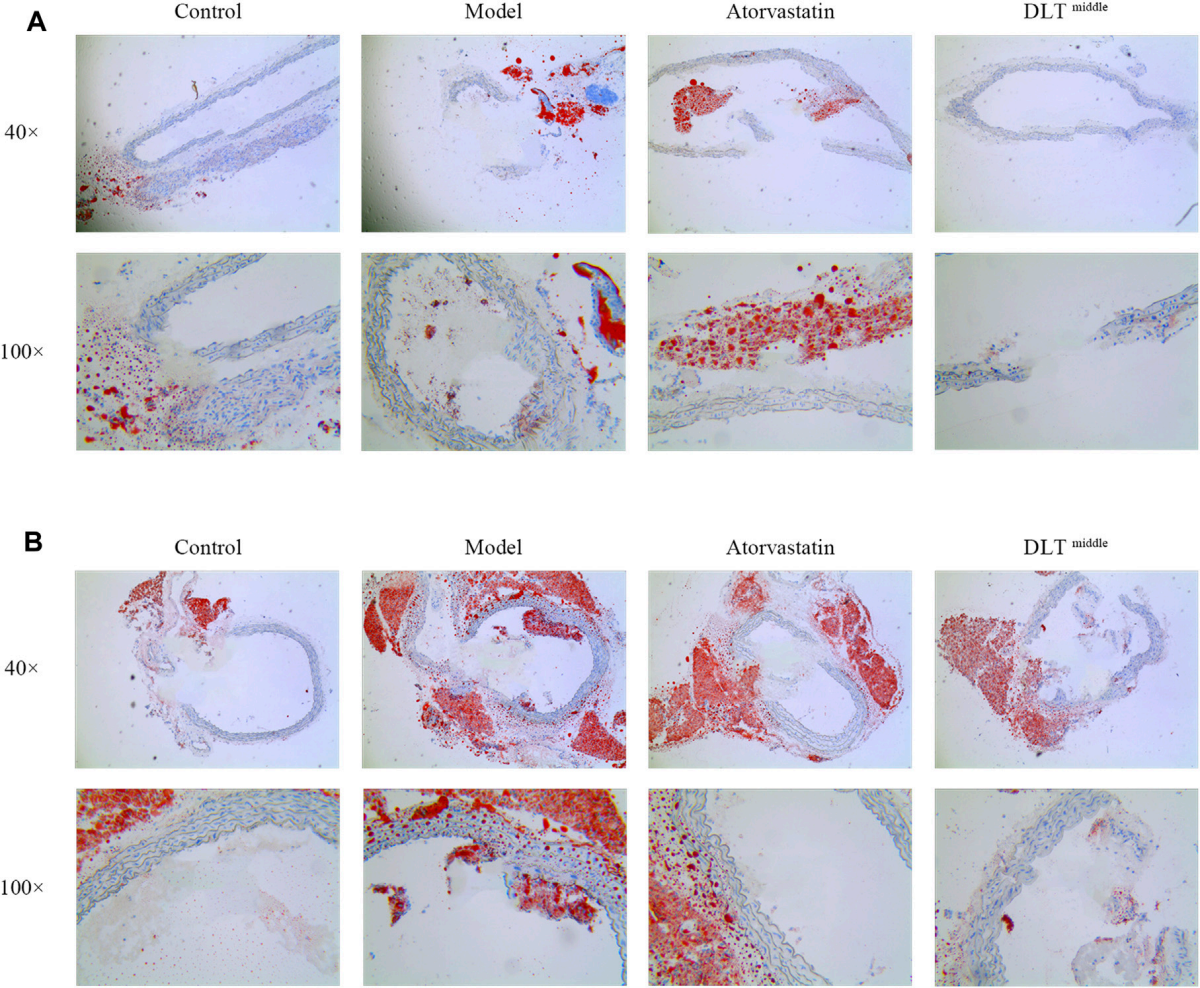
DLTs reduced intracellular lipid droplet accumulation in the aorta and left carotid artery of ApoE^-/-^ mice. Intracellular lipid droplet accumulation was observed by oil red O staining in the carotid **(A)** and aortic **(B)** arteries. Images revealed the most severe intracellular lipid droplet deposition (in red) in the vessel wall of the model groups, while it was markedly reduced in the DLT-middle group in aortic tissue and carotid tissue compared with the model group, so did the atorvastatin group in aortic tissue. No significant difference in lipid droplet deposition was seen between the atorvastatin group and the model group in carotid tissue.

In addition, oil red O-stained areas in sections of the left carotid and aorta artery tissue in the model groups were larger than them in the control groups. The administration of middle-dose DLTs markedly decreased the area of lipid deposition in the left carotid and the aorta vessel. Similarly, lipid droplet accumulation was reduced in aortic tissue in the atorvastatin group compared with the model group whereas there was no difference in the accumulation of lipid droplets in the left carotid artery tissue between two groups (Figure 2).

### Immunohistochemical Observation of Macrophage (CD68) Infiltration, Inflammatory Molecule IFN-γ and Vascular Smooth Muscle (α-actin) in the Aorta of ApoE^−/−^ Mice

**FIGURE 3:**
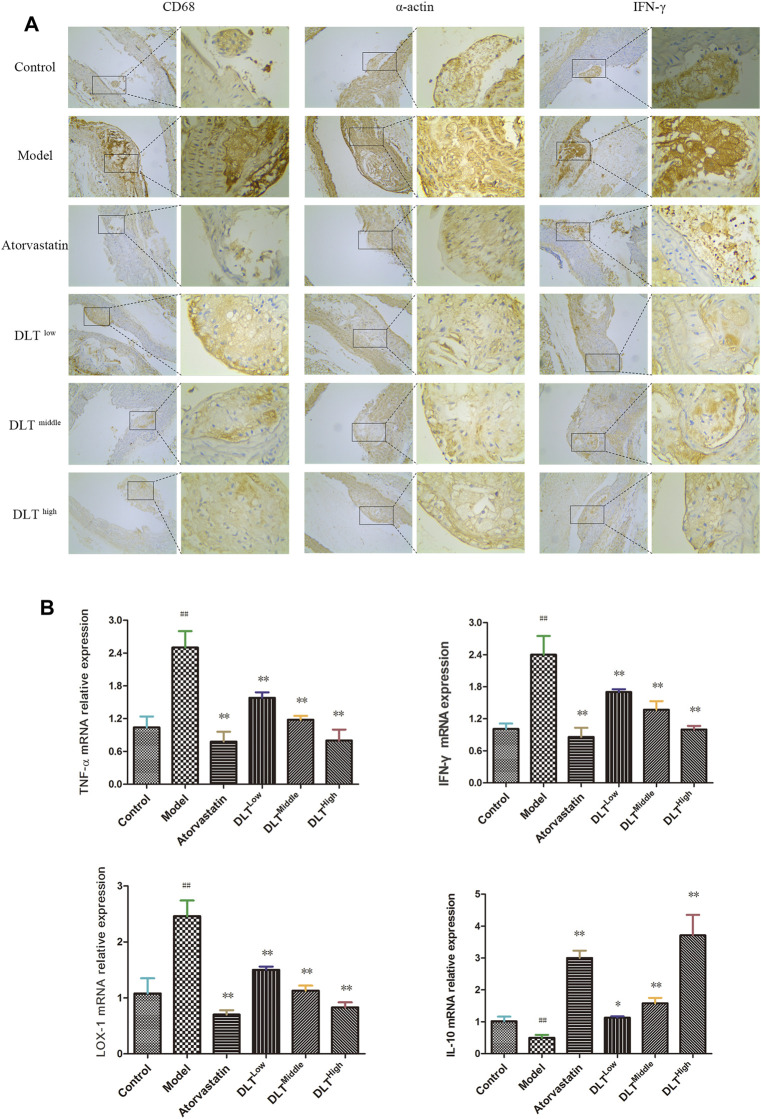
Changes in macrophage (CD68) infiltration and the expression of LOX-1, the inflammatory factors TNF-α, IFN-γ, IL-10 and smooth muscle (α-actin) in the aorta of ApoE^-/-^ mice. Macrophage (CD68) infiltration, the inflammatory molecule IFN-γ and smooth muscle (α-actin) were evaluated by immunohistochemical staining **(A)**, and the mRNA expression levels of LOX-1, TNF-α, IFN-γ and IL-10 were analyzed by RT-PCR (B). **(A)** CD68, α-actin, and IFN-γ immunohistochemical staining showed brown-yellow granules atherosclerotic lesions of the aorta in each group. Among all groups, the brown-yellow granules in the model group were the most abundant and were decreased gradually in a dose-dependent manner in the DLT groups; the atorvastatin group also exhibited fewer yellow granules compared with the model group. **(B)** mRNA levels of the proinflammatory factors IFN-γ and TNF-α and oxidized low-density lipoprotein receptor LOX-1in the model group were significantly higher than those in the control group. The atorvastatin group and the DLT groups treated with low, middle, and high doses exhibited reduced expression of IFN-γ, TNF-α and LOX-1 mRNA. The mRNA expression of the anti-inflammatory factor IL-10 was significantly increased in the atorvastatin group and DLT groups. The data are presented as the mean ± SEM (*n* = 7). ^##^ *p* < 0.01 vs the control group, and ^*^ *p* < 0.05, ^**^ *p* < 0.01 vs the model group.

Under a light microscope, CD68, α-actin and IFN-γ (brown-yellow particles) were observed in the atherosclerotic lesions of the aorta (Figure 3A). Compared with the control group, the expression of CD68, IFN-γ and α-actin in the atherosclerotic plaques of ApoE^−/-^ mice was significantly increased in the model group; compared with the model group, the expression of CD68, IFN-γ and α-actin in the atherosclerotic plaques of the aorta in the DLT groups of different doses were reduced in a dose-dependent manner. Similarly, the expression of CD68, IFN-γ and α-actin in ApoE^−/-^ mice in the atorvastatin group was markedly reduced, suggesting that DLTs inhibited macrophage (CD68) infiltration, the expression of inflammatory molecule IFN-γ and vascular smooth muscle (α-actin) in the aorta of ApoE^−/-^ mice fed with a high-fat diet.

### Effect of Danlou Tablets on the mRNA Expression of IFN-γ, TNF-α, LOX-1 and IL-10 in the Atherosclerosis Model of ApoE^-/-^ Mice

To observe the effects of DLTs on the expression of pro- and anti-inflammatory factors, as well as lipid metabolism in vascular tissue, the expressions of the inflammatory factors IFN-γ, TNF-α (proinflammatory) and IL-10 (anti-inflammatory) and LOX-1 were determined by real-time PCR in aortic tissues of mice in each group (Figure 3B). The results showed that the IFN-γ, TNF-α and LOX-1 mRNA levels of the model group were significantly higher than those of the control group (*p* < 0.01). Atorvastatin and DLTs at low, middle, and high doses reduced the mRNA expression of IFN-γ, TNF-α and LOX-1 (*p* < 0.01). The expression of IL-10 mRNA was significantly increased in the atorvastatin group and DLT groups vs the model group (*p* < 0.05 or *p* < 0.01).

### Changes in Blood Lipid Levels of ApoE^−/-^ Mice

**FIGURE 4:**
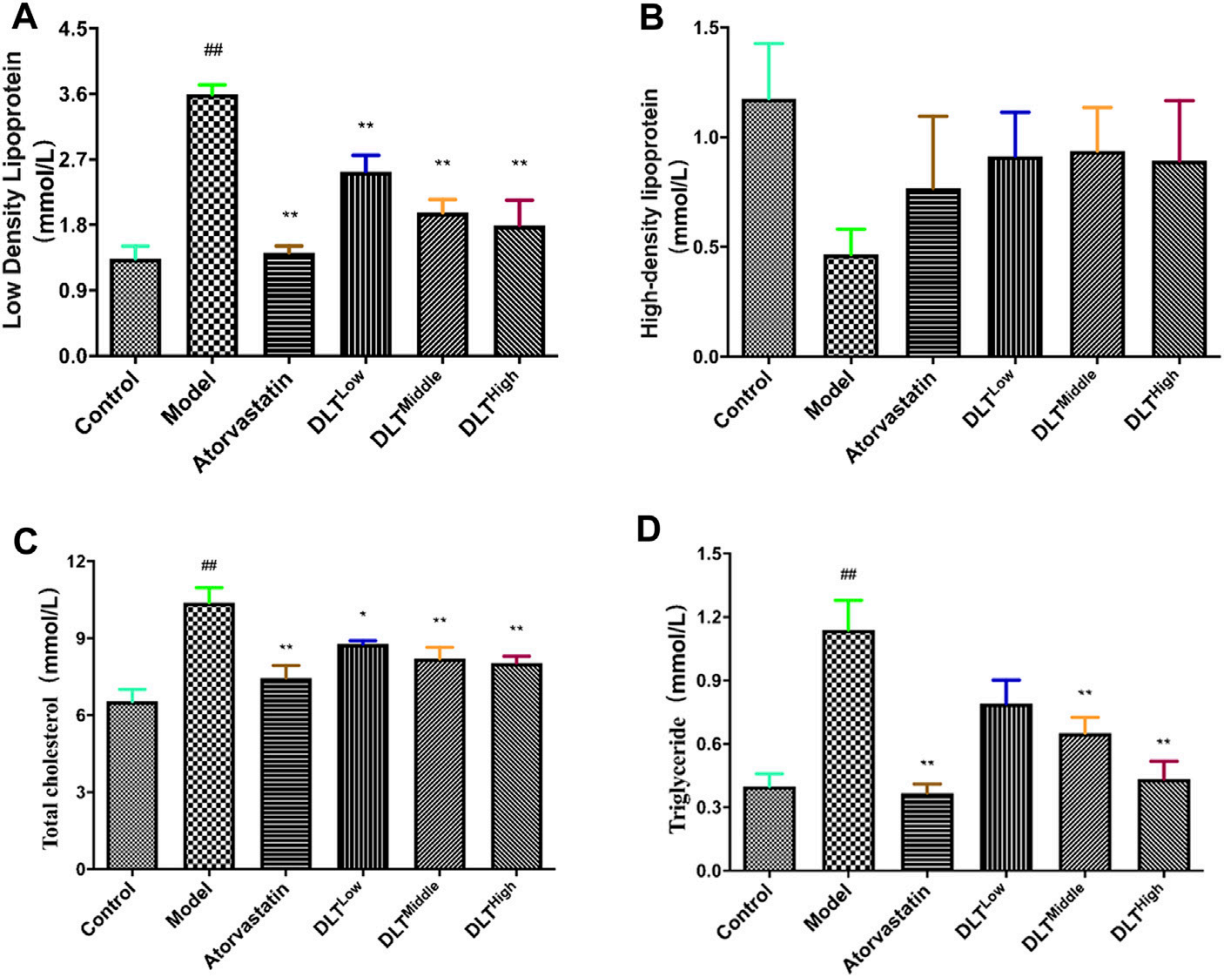
DLTs influenced plasma lipids following a 16 week high-fat diet in AS APOE^-/-^ mice. **(A)** Low-density lipoprotein. **(B)** High-density lipoprotein. **(C)** Total cholesterol. **(D)** Triglyceride. The data are presented as the mean ± SD (*n* = 7). ^*##*^ *p* < 0.01 vs the control group, and ^*^ *p* < 0.05, ^**^ *p* < 0.01 vs the model group.

Serum lipid levels were measured at the end of the experiment. The results showed that the serum LDL-C, TC, and TG levels of the model group were increased compared with those of the control group (*p* < 0.01). In terms of HDL-C levels, no difference was observed among all groups (*p* > 0.05). Significant differences were respectively observed in the levels of LDL-C and TC between the low-, middle-, and high-dose DLT groups and the model group. It was indicated that compared with the model group, DLT decreased the serum levels of LDL-C and TC (*p* < 0.05 or *p* < 0.01). As for the serum level of TG, there is no significant difference between low-dose DLT group and the model group (*p* > 0.05). However, in the middle- and high-dosage of DLT groups, TG levels were sharply lower than those of the model group (*p* < 0.01). The serum levels of LDL-C, TC and TG in the positive control group (atorvastatin) mice were significantly lower than those in the model group (*p* < 0.01). Experiments showed that the blood lipid levels of mice in each group of DLTs gradually decreased in a dose-dependent manner (Figure 4).

### Network Pharmacology Results

**FIGURE 5:**
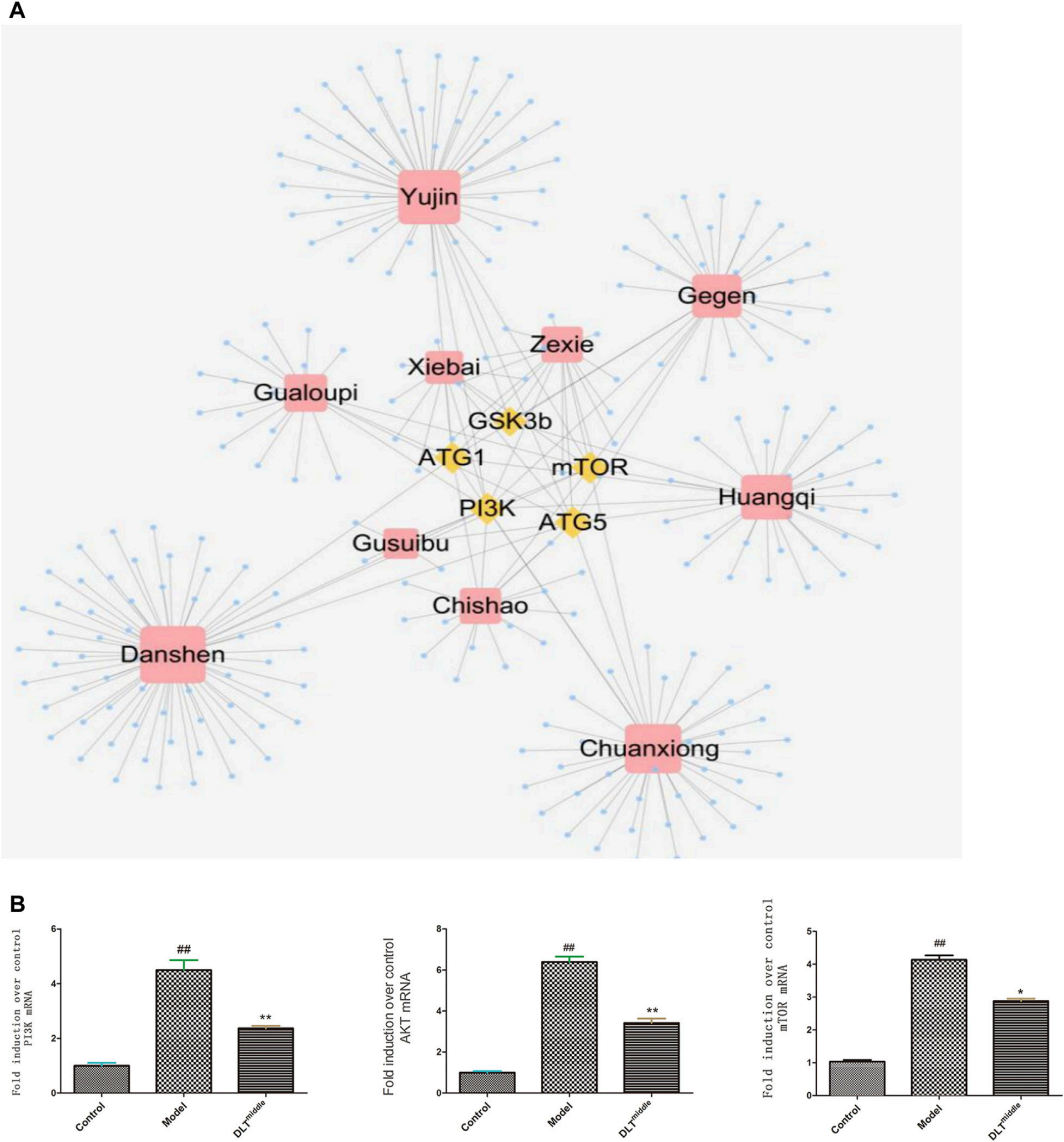
DLTs regulated autophagy through the PI3K/Akt/mTOR signaling pathway in AS APOE^-/-^ mice. **(A)** Compound-target network for DLTs. The yellow nodes represent potential autophagy-related targets, and the blue nodes represent active compounds. **(B)** mRNA expression of PI3K/AKT/mTOR in aorta tissues. The data are presented as the mean ± SD (*n* = 3). ^##^ *p* < 0.01 vs the control group, and ^*^ *p* < 0.05, ^**^ *p* < 0.01 vs the model group.

Active ingredients in DLTs were preliminarily screened according to “Lipinski’s rule of five” and drug-likeness (DL) ≥ 0.18 criteria. The component-target network indicated that five target genes-GSK3b, ATG1, ATG5, PI3K and mTOR in autophagy pathways were closely associated with 253 active components of DLTs after molecular docking (Table 2). The results of the molecule-herb-target network are shown in Figure 5A. The RT-PCR results showed that the mRNA levels of PI3K, Akt and mTOR were upregulated in the aortic tissues of ApoE^−/-^ mice (*p* < 0.01) and were downregulated by the middle doses of DLTs (*p* < 0.01) (Figure 5B), which suggested that DLTs were involved in regulating the PI3K/Akt/mTOR pathway to enhance autophagy.

**TABLE 2:** The number of active ingredients in herbs of DLTs and their main autophagy-related targets.

Herbs	Number of active ingredients	Target genes
Danshen	59	ATG1, ATG5, mTOR, PI3K
Yujin	48	GSK3b, ATG1, ATG5, mTOR, PI3K
Chuanxiong	39	ATG1, ATG5, mTOR, PI3K
Huangqi	31	GSK3b, ATG1, ATG5, mTOR, PI3K
Gegen	26	GSK3b, ATG1, ATG5, mTOR, PI3K
Gualoupi	16	ATG1, ATG5, mTOR, PI3K
Chishao	13	ATG1, ATG5, mTOR, PI3K
Zexie	12	GSK3b, ATG1, ATG5, mTOR, PI3K
Xiebai	6	GSK3b, ATG1, ATG5, mTOR, PI3K
Gusuibu	3	ATG5, mTOR, PI3K

### Danlou Tablets Inhibited the Growth of RAW264.7 Cells

**FIGURE 6:**
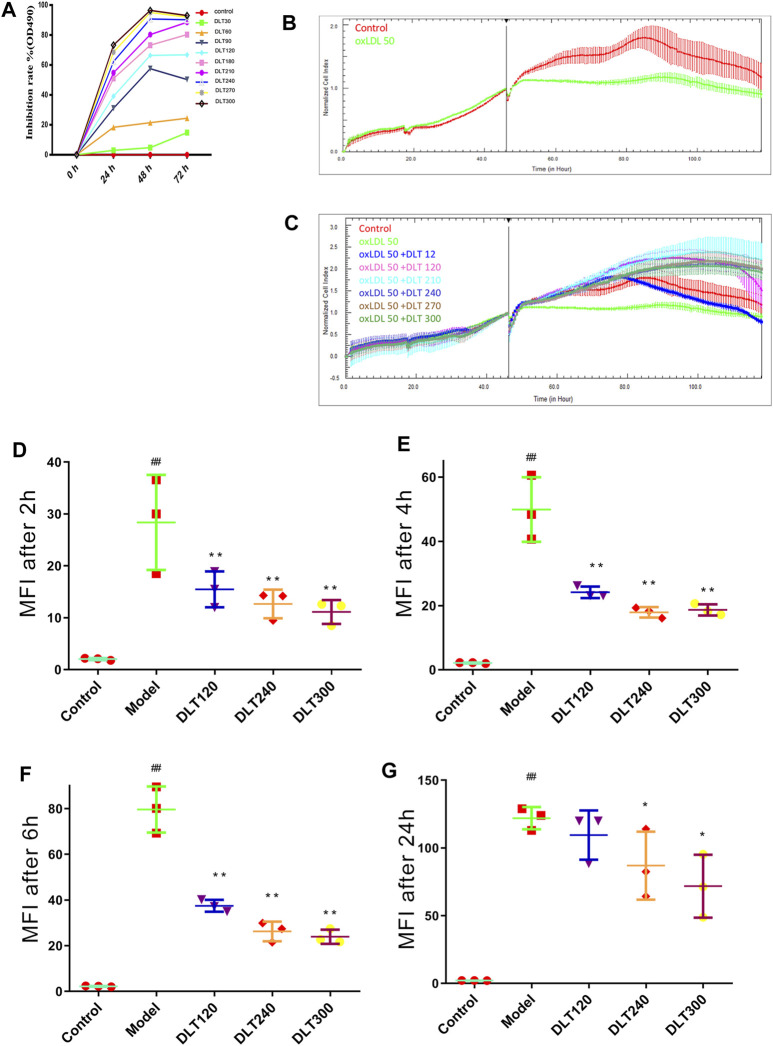
DLTs affected the viability and phagocytosis of Dil-oxLDL in RAW264.7 cells. **(A)** Viability of RAW264.7 cells treated with DLTs. **(B)** CI of RAW264.7 cells treated with ox-LDL. **(C)** CI of RAW264.7 cells treated with DLTs and ox-LDL. **(D–G)** The extent of Dil-oxLDL uptake (MFI) by RAW264.7 cells after treatment with DLTs for 2, 4, 6 and 24 h was determined by flow cytometry, and the results are shown as bar graphs. The data are presented as the mean ± SD, ^##^ *p* < 0.01 vs the control group, and ^*^ *p* < 0.05, ^**^ *p* < 0.01 vs the model group.

The MTT assay was performed to evaluate the inhibitory effects of DLTs on RAW264.7 cells. As shown in Figure 6A, DLT concentrations from 30 to 300 μg/ml significantly inhibited the proliferation of RAW264.7 cells at different time points (24, 48, and 72 h) in a dose-dependent manner (*p* < 0.01).

### Danlou Tablets Increased the Cell Index of RAW264.7 Cells Stimulated by oxLDL

The xCELLigence system was used to evaluate the effect of DLT concentration on RAW264.7 cells transforming to foam cells induced by 50 μg/ml oxLDL. The cell index of the model group was lower than that of the control group, indicating that foam cells could cause cell index decline. In addition, the cell index decreased after 80 h of cell inoculation (Figure 6B). The cell index of the DLT groups at different concentrations was significantly higher than that of the model group, which indicated that DLTs significantly inhibited the formation of foam cells. The cell growth index curve was higher than that of the control group when the DLT concentration increased; indicating that DLTs could enhance the oxLDL-induced growth activity of RAW264.7 cells (Figure 6C).

### Danlou Tablets Inhibited RAW264.7 Macrophage Phagocytosis of oxLDL

The median fluorescence intensity (MFI) was measured by flow cytometry to analyze the content of Dil-oxLDL in RAW264.7 macrophages. After treatment with Dil-oxLDL and various concentrations of DLTs, the cells were collected at 2, 4, 6 and 24 h for flow cytometry analysis. After 2 h of incubation, the MFI values of the DLT groups treated with 60–300 μg/ml DLTs were markedly lower than those of the model group (*p* < 0.05) (Figures 6D–F). 24 h later, the MFI of the groups treated with 240 and 300 μg/ml DLTs was significantly decreased (*p* < 0.05) (Figure 6G).

### Danlou Tablets Alleviated the Apoptosis of BMDMs Induced by Ox-LDL

**FIGURE 7:**
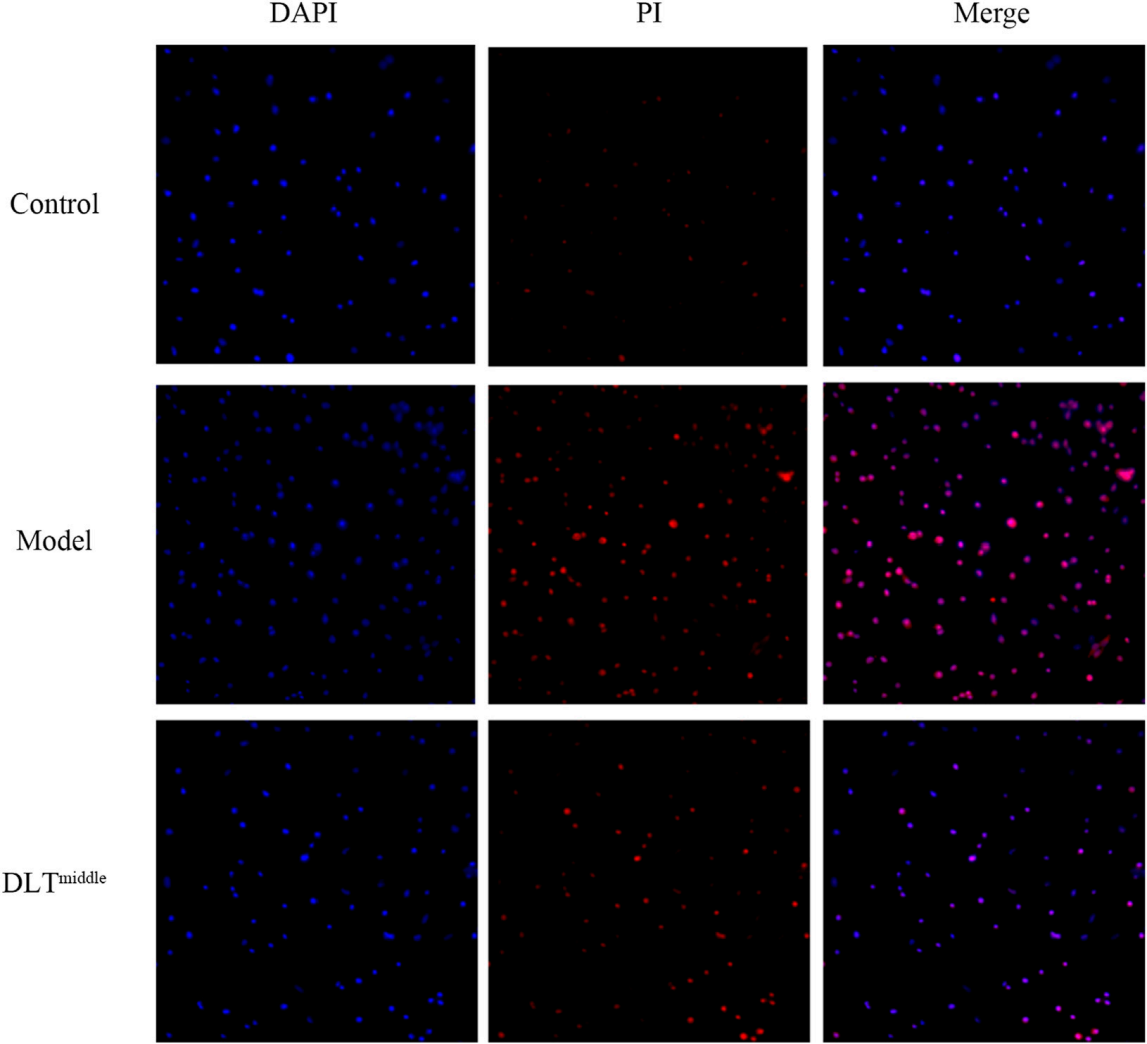
DLTs suppressed apoptosis of BMDMs *in vitro*. Apoptotic cells were stained with DAPI and PI and observed by fluorescence microscopy (magnification ×100) after treatment with ox-LDL and DLT^middle^. More cells with deepened nuclear staining (merge) were seen in the ox-LDL-induced BMDM group than in the control group, cells with deepened nuclear staining were significantly reduced after DLT treatment.

To examine the effects of DLTs on ox-LDL-induced apoptosis in BMDMs, we stained the cells in each group with DAPI and PI to observe the morphology of the nuclei under an immunofluorescence microscope (Figure 7). Apoptotic cells showed nuclear condensation with deepening of staining or nuclear fragments. The results showed more cells with deepened nuclear staining in the ox-LDL-induced BMDM group than in the control group, and the DAPI-PI dye-treated cells were significantly reduced after DLT treatment.

### Danlou Tablets Might Alleviate Intracellular Lipid Accumulation Through Autophagy

**FIGURE 8:**
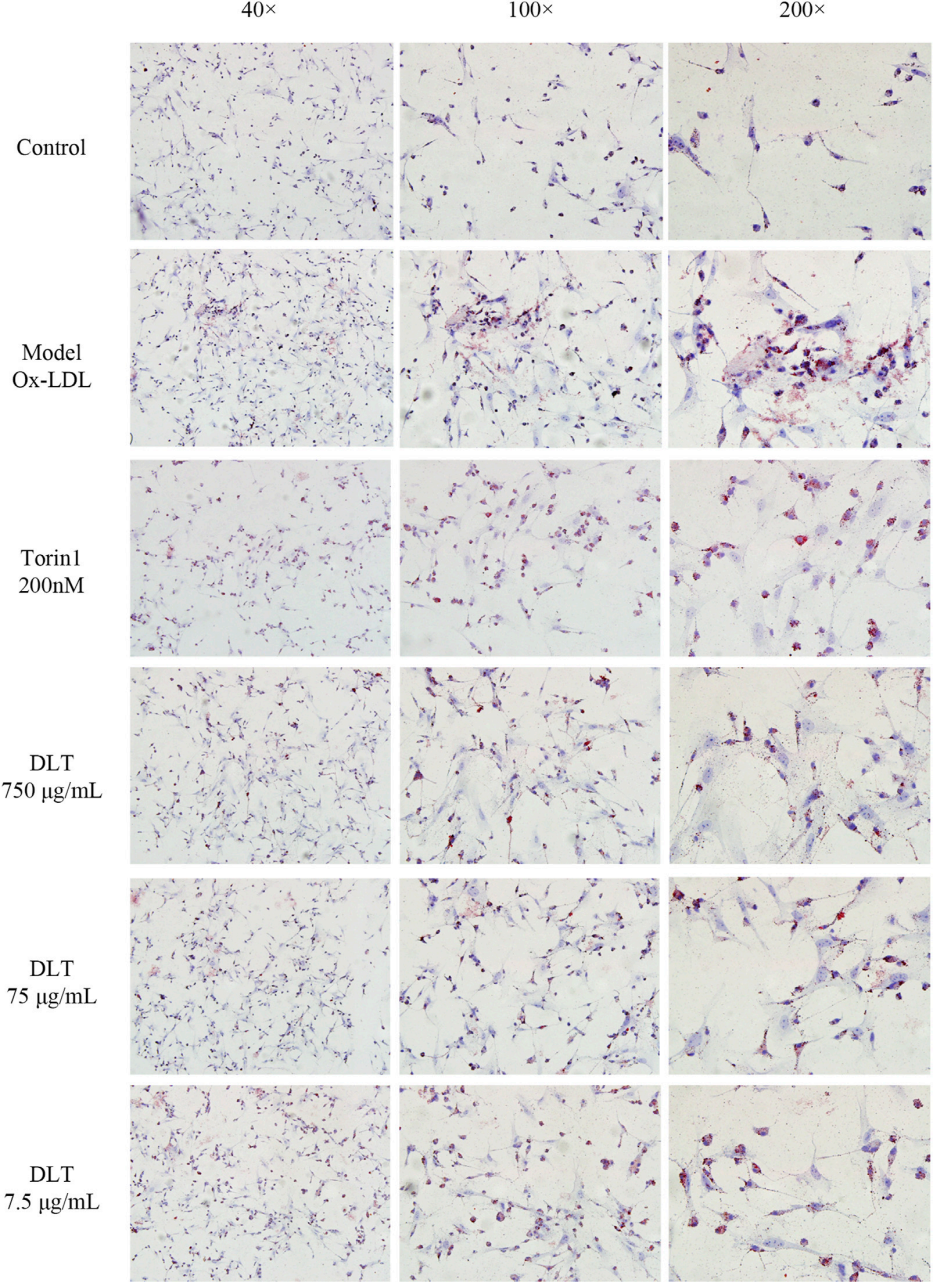
DLTs reduced the accumulation of intracellular lipids in BMDMs. Representative images of oil red O staining are presented (magnification ×40, ×100, ×200). Ox-LDL-induced BMDM group showed more red lipid droplets, while the Torin 1 group and the DLT groups exhibited less red lipid droplets.

**FIGURE 9:**
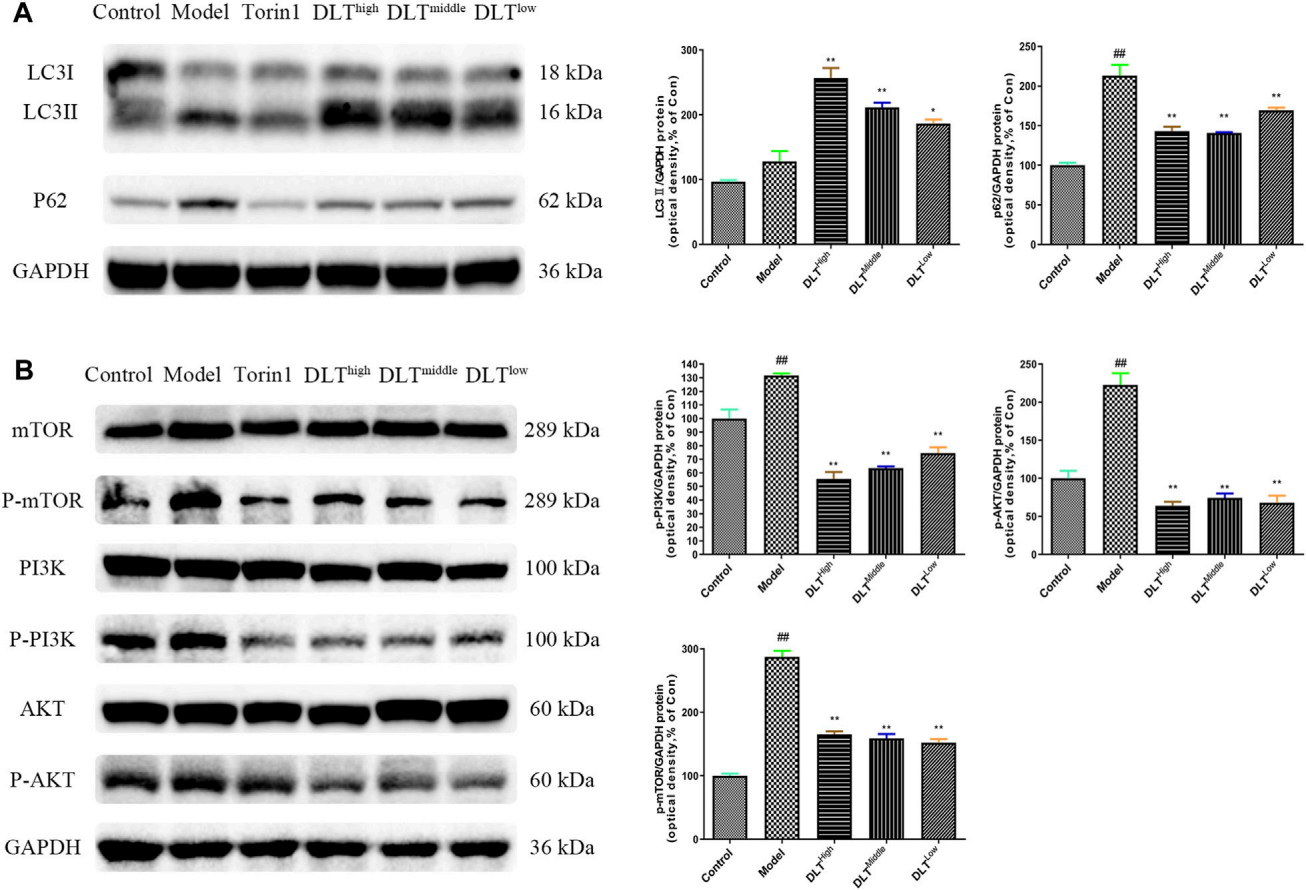
DLTs enhanced autophagy in oxLDL-induced RAW264.7 cells via the PI3K/Akt/mTOR pathway. **(A)** DLTs upregulated the expression of the autophagy-related protein LC3II and decreased P62 accumulation. **(B)** DLTs downregulated the expression of the autophagy-related signaling molecules p-PI3K, p-Akt, and p-mTOR. The bar graphs show the quantification of the indicated proteins. Mean ± SD, *n* = 3. ^##^ *p* < 0.01 vs the control group, and ^*^ *p* < 0.05, ^**^ *p* < 0.01 vs the model group.

To observe the inhibitory effects of autophagy on BMDMs transformation into foam cells, oil red O staining was performed, and the differences between Torin1 and DLT treatments were compared. Torin1 is an inhibitor of mTOR, which is a key molecule inducer of autophagy. Our results showed that Torin1 treatment significantly reduced intracellular lipid droplet accumulation as did DLT treatment; that is, promoting autophagy also inhibited macrophage transition into foam cells (Figure 8). Moreover, the expression of the autophagy indicators LC3II and P62 suggested enhanced autophagy in DLT-treated RAW264.7 Macrophages induced by oxLDL (Figure 9A).

### Danlou Tablets Might Alleviate Intracellular Lipid Accumulation Through an Autophagy-Related PI3K/Akt/mTOR Signaling Pathway in RAW264.7 Cells

Because our network pharmacology results indicated that DLTs affected PI3K, ATG5, ATG1 and mTOR targets, we evaluated whether the PI3K/Akt/mTOR pathway was involved in the regulation of DLTs in RAW264.7 cells induced by oxLDL. The data showed that similar to Torin1, p-PI3K, p-Akt and p-mTOR were inhibited in DLT-treated RAW264.7 cells (Figure 9B), which suggested that DLTs inhibited the autophagy-related PI3K/Akt/mTOR signaling pathway to alleviate intracellular lipid accumulation in RAW264.7 cells.

## Discussion

Atherosclerosis is a progressive disease that occurs in the intima of large and medium-sized arteries (Ahmed, Ameen et al., 2021); it is characterized by the buildup and fusion of scattered plaques in the arterial intima. Dyslipidemia is the main risk factor for atherosclerosis (Ma, Leng et al., 2019). Macrophage uptake and phagocytosis of lipid proteins, such as ox-LDL, result in the accumulation of intracellular cholesterol ester and transformation into foam cells, which is a key link in the progression and outcome of atherosclerosis (Orekhov, Nikiforov et al., 2020).

Traditional Chinese medicine (TCM) holds that the basic disease mechanism of atherosclerosis is a deficiency in origin and excess in superficiality with phlegm and blood stasis. Therefore, “cotreatment of phlegm and stasis” would be an effective treatment for atherosclerosis. DLTs are a representative prescription for the “cotreatment of phlegm and blood stasis”, which has been approved by the national CFDA for clinical use and has achieved good clinical efficacy.

Knockout of the ApoE gene in mice caused low-density lipoprotein metabolism disorders with low-density lipoproteins oxidized and deposited in blood vessels, ultimately leading to severe hyperlipidemia and atherosclerotic lesions (Bobryshev 2006; Li Y. et al., 2016). In this study, ApoE^−/-^ mice were fed a high-fat diet for 16 weeks to establish an atherosclerosis model, and the effects of DLTs on the occurrence and development of atherosclerosis were observed. The results showed that DLTs significantly decreased the serum levels of TC, TG, and LDL in AS mice and inhibited the formation of atherosclerotic plaques, arterial intimal hyperplasia and foam cell formation in a dose-dependent manner. These results indicated good antiatherosclerotic effects of DLTs.

Atherosclerosis is also a chronic inflammatory disease affecting the aorta. Inflammatory cytokines, such as TNF-α and IFN-γ, have a role in enhancing VCAM-1 expression and monocyte recruitment to the vessel wall (Zhang, Alcaide et al., 2011; Lee, Ha et al., 2020) and mediating endothelial cell death (Pober and Sessa 2007). IFN-γ signaling mediates the upregulation of LDL scavenger receptor expression and therefore enhances foam cell formation. Both TNF-α and IFN-γ resulted in the remarkably increased accumulation of subintimal macrophages and accelerated atherosclerosis. While IL-10 inhibits monocytes to release proinflammatory cytokines, including IL-1β, TNF-α, and IL-8, and promotes macrophage polarization toward the M2 phenotype (Han and Boisvert 2015), it inhibits the expression of metalloproteinase (MMP), proinflammatory cytokines and cyclooxygenase-2 in lipid-engulfed activated macrophages. In our study, the mRNA levels of TNF-α and IFN-γ were abundantly expressed in the atherosclerotic lesions of the aorta in atherosclerosis mice, while the levels of IL-10 were the opposite. DLTs reversed this trend.

Foam cell formation plays a pivotal role during atherogenesis and progression (Yu, Fu et al., 2013; Maguire, Pearce et al., 2019). Macrophages phagocytose large amounts of oxLDL *via* a scavenger receptor (SR) on their surface, resulting in the formation of foam cells (Bobryshev 2006), which induce a vascular local inflammatory response that exacerbates plaque formation. Macrophagic foam cell formation is thought to be the result of an imbalance in intracellular lipid metabolism, i.e., cholesterol influx and efflux (Sukhorukov and Khotina, 2020). Our results showed that DLTs could obviously alleviate macrophage transformation into foam cells (MFI), accumulation of lipid droplets (oil red O staining) and infiltration (CD68) in arterial tissues.

The role of autophagy in macrophage lipid metabolism has been highlighted by many researchers (Sun, Fan et al., 2018; Cao, Jia et al., 2019; Zhou, Ren et al., 2019). MD Khurshidul Zahid et al. found that enhancing autophagy could drive cholesterol efflux and inhibit macrophagic foam cell formation in RAW264.7 cells (Zahid, Rogowski et al., 2020). Liu et al. proved that the autophagy activator rapamycin markedly decreased intracellular lipid content and prevented transformation into foam cells, while the autophagy inhibitor 3-MA considerably increased intracellular lipid droplet accumulation. *In vivo* experiments showed that rapamycin administration in ApoE^−/−^ mice reduced the death rate of macrophages and delayed plaque progression (Liu, Tang et al., 2016).

To further explore the mechanism of DLTs acting on atherosclerosis mice, we established a model of BMDMs induced by oxLDL *in vitro* and observed the effects of autophagy on foam cell formation, as well as autophagy-related pathways involved in the regulation of DLTs on BMDMs. The results showed that lipid droplet accumulation within macrophages can be reduced by promoting autophagy when using the autophagy activator Torin1. Enhanced autophagy by DLTs was confirmed by Western Blotting, which showed enhanced LC3Ⅱ expression and attenuated p62 accumulation. In addition, based on the network pharmacology results of DLTs with autophagy, as well as the animal experimental results, we also validated that DLTs regulated autophagy *via* the PI3K/Akt/mTOR signaling pathway in oxLDL-induced RAW264.7 cells.

In short, we conducted systematic research on DLTs, including component analysis, *in vivo* and *in vitro* studies, and network pharmacological target prediction. We confirmed that DLTs indeed ameliorated atherosclerosis in ApoE^−/−^ mice. In addition, we explored the mechanism of DLTs in atherosclerosis. DLTs may promote autophagy by inhibiting the PI3K/Akt/mTOR signaling pathway, thereby reducing the uptake of ox-LDL by macrophages, which in turn results in the formation of foam cells and the inhibition of atherosclerosis.

## Data Availability

The original contributions presented in the study are included in the article/Supplementary Material, further inquiries can be directed to the corresponding authors.

## Supplementary Material

Supplementary Figure S1 UHPLC fingerprints of 5 batches of DLT. 2:danshensu; 9:Salvianolic acid A; 10:Cryptotanshinone; 11:tanshinone ⅡA.
