# Supplementary figures and images for: Danlou Tablets Inhibit Atherosclerosis in Apolipoprotein E-Deficient Mice by Inducing Macrophage Autophagy: The Role of the PI3K-Akt-mTOR Pathway
Source: Front Pharmacol. 2021 Sep 8;12:724670. doi: 10.3389/fphar.2021.724670 (PMC8455997; doi:10.3389/fphar.2021.724670)

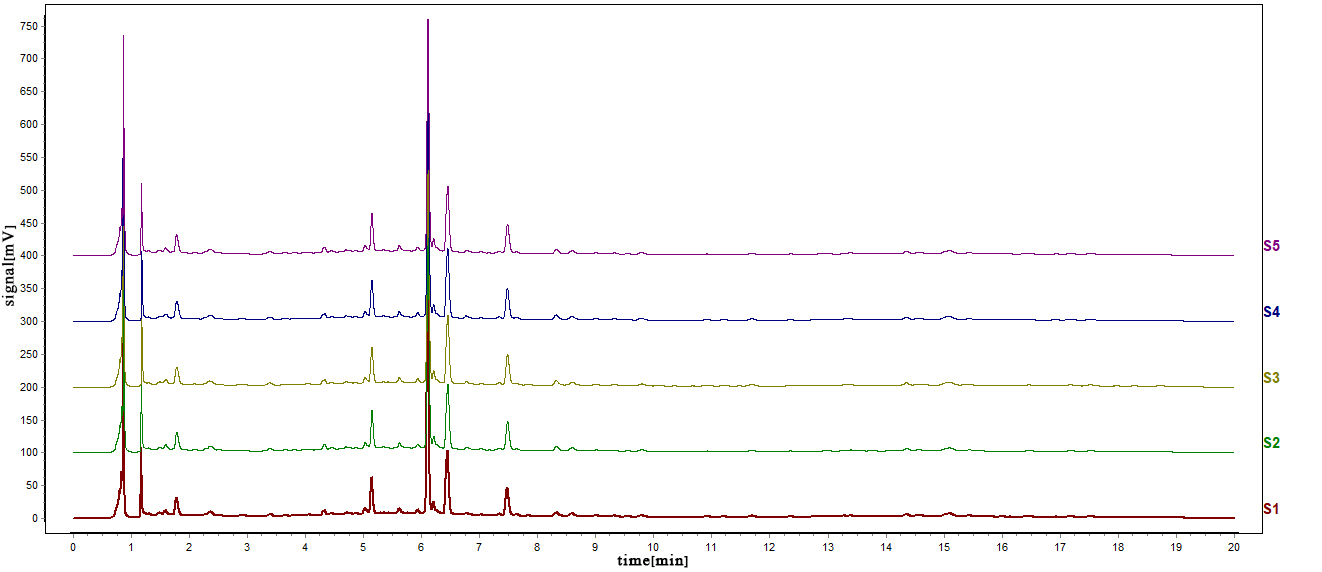

Supplement: Supplementary file 1 [file Image1.jpg]
